# Patient assessment of a novel therapeutic approach for the treatment of severe, chronic pain

**DOI:** 10.1111/j.1742-1241.2008.01820.x

**Published:** 2008-08

**Authors:** J Nadstawek, P Leyendecker, M Hopp, C Ruckes, S Wirz, W Fleischer, K Reimer

**Affiliations:** 1 Clinic of Anaesthesiology and Intensive Care MedicineUniversity of Bonn, Bonn, Germany; 2 Mundipharma Research GmbH & Co. KGLimburg (Lahn), Germany; 3 University Witten/HerdeckeWitten, Germany

## Abstract

**Background and objectives:**

Opioid-induced constipation can have a major negative impact on patients’ quality of life. This randomised clinical trial evaluated patient assessment of the efficacy and tolerability of oral prolonged-release (PR) oxycodone when co-administered with oral naloxone PR.

**Methods:**

Two hundred and two patients with chronic cancer- or non-cancer-related pain undergoing stable oxycodone PR therapy (40, 60 or 80 mg/day) were randomised to one of four intervention groups: 10, 20 or 40 mg/day naloxone PR or placebo. Following a 4-week maintenance phase, patients were followed-up for 2 weeks in which time they received oxycodone PR only. At the end of the maintenance phase, patients and investigators were asked to assess treatment efficacy and tolerability, as well as preference for the titration or maintenance phase.

**Results:**

Patient and investigator global assessment of efficacy and tolerability improved with increasing naloxone dose. Efficacy was ranked as ‘good’ or ‘very good’ by 50.0%, 67.4% and 72.5% of patients in the 10, 20 and 40 mg naloxone PR dose groups, respectively, compared with 43.5% of patients in the placebo group. Patient assessment of tolerability was similar between treatment groups and placebo, being ranked as ‘good’ or ‘very good’ by 83.3%, 79.1% and 82.5% of patients in the 10, 20 and 40 mg/day naloxone PR dose groups, respectively, compared with 71.7% of patients in the placebo group. The maintenance treatment phase was preferred by patients in the naloxone groups. A 2 : 1 dose ratio of oxycodone to naloxone was also assessed. Efficacy was ranked as ‘good’ or ‘very good’ by 70.4% of patients treated with the 2 : 1 dose ratio compared with 43.5% of patients receiving placebo. Tolerability of the 2 : 1 dose ratio was ranked as being ‘good’ or ‘very good’ by 81.5% of patients compared with 71.1% for the placebo group and patients preferred the maintenance phase.

**Conclusions:**

The co-administration of oral naloxone PR with oxycodone PR improves patient assessment of analgesic opioid therapy for severe chronic pain, in terms of both efficacy and tolerability.

**What's known**

Constipation is the most commonly reported adverse event associated with opioid use. Opioid-induced constipation causes significant discomfort, severely affecting patients' quality of life and forcing patients either to discontinue their opioid therapy or reduce opioid dose, resulting in inadequate pain control.

**What's new**

Co-administration of oral oxycodone PR and the opioid antagonist oral naloxone PR provides effective analgesia while reducing the symptoms of opioid-induced constipation. Patient perception of their analgesic therapy is improved by the co-administration of oral oxycodone PR and naloxone PR in terms of both efficacy and tolerability.

## Introduction

Oxycodone is a strong, semi-synthetic opioid (1) that has been in clinical use since 1917 for the treatment of moderate-to-severe chronic pain (2). It has demonstrated efficacy in the treatment of postoperative, osteoarthritis and cancer-related pain (1,3,4). Oxycodone is also effective in the treatment of the different syndromes of neuropathic pain, such as diabetic neuropathy (5,6) and postherpetic neuralgia (7).

The opioid-mediated side effects of opioid therapy are well characterised and include respiratory depression, nausea, sedation, euphoria or dysphoria, constipation and itching (8). Constipation is the most frequently reported adverse event associated with chronic opioid therapy (9). It is just one of a number of symptoms of opioid-induced bowel dysfunction (OBD), which can also include hard dry stools, straining, bloating, abdominal cramping, distension and increased gastric reflux (10). While many of the side effects associated with opioid therapy resolve with long-term use, no tolerance appears to occur for constipation (8). The physical discomfort and pain caused by constipation can force patients either to discontinue their opioid therapy (11) or reduce the opioid dose, resulting in inadequate pain control. As a consequence, constipation is an important adverse event that requires treatment.

Laxative regimens are established for clinical use both for prophylaxis and treatment of opioid-induced constipation. However, they are non-specific, as they do not affect the opioid receptor-mediated reason for constipation, and are often ineffective (10,11).

The adverse effect of opioids on bowel function stem largely from binding to opioid receptors in the plexus myentericus and plexus submucosus of the gut, while the analgesic effects are largely due to μ-opioid receptor binding in the central nervous system (11,12). It should therefore be possible to separate these two effects to provide analgesia with reduced opioid-induced constipation.

Naloxone is a competitive (μ, δ and κ) opioid receptor antagonist that is mainly used intravenously to reverse opioid overdose because of its high receptor affinity (13). Because of extensive first-pass hepatic metabolism, orally administered naloxone has negligible systemic bioavailability of approximately 2% (14). At therapeutic oral doses, naloxone exerts a local inhibitory effect on opioid action in the gastrointestinal system without interfering with the central nervous system. The administration of oral naloxone may therefore reduce opioid-induced constipation (and other aspects of OBD), while allowing the centrally mediated analgesic effect of opioids.

A Phase II trial was conducted to assess the analgesic efficacy of prolonged-release (PR) oxycodone in combination with orally administered naloxone PR in patients with severe, chronic pain, and to evaluate the efficacy of the combination in improving bowel function. One of the secondary endpoints of the trial involved evaluating the patients' and investigators' preference for treatment. The analgesic efficacy and bowel function results of the trial have been published separately. Co-administration of oxycodone PR and naloxone PR provided effective analgesia while significantly reducing the symptoms of OBD (15). The present paper reports the findings on patient and investigator global assessment of efficacy, tolerability and treatment preference.

## Methods

This was a multicentre, prospective, placebo-controlled, randomised, double-blind, parallel-group Phase II trial conducted in 28 centres in Germany from May 2002 to April 2003. The study was conducted in accordance with the Declaration of Helsinki and its successors (Edinburgh 2000 and Washington 2002) and complied with the principles of Good Clinical Practice set by the International Conference on Harmonization and applicable German regulatory requirements. Written informed consent was obtained from patients at screening.

Male and female patients aged 18 years and over were eligible to enter the study if they had severe cancer or non-cancer pain requiring opioid treatment and/or insufficient efficacy or tolerability with a WHO II or III analgesic, or were under stable oxycodone therapy (40–80 mg/day). Exclusion criteria included current alcohol or drug abuse, severe cardiovascular or respiratory disease, severe liver and renal insufficiency and/or liver/renal carcinoma and/or metastases. Patients were also excluded if they had a history of paralytic ileus, psychoses or Parkinson's disease, current acute pancreatitis, were taking early disease-related retirement, receiving opioid treatment beside oxycodone, had a known hypersensitivity to one of the study drugs, or had participated in another clinical trial within 30 days. Female patients who were of childbearing age, but not adequately protected against conception, or who were pregnant or lactating were also excluded.

**Figure 1:**
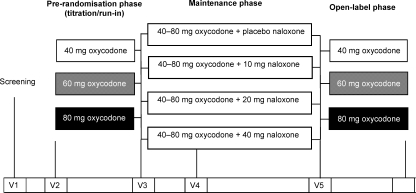
Study design. V = visit. Oxycodone and naloxone are prolonged-release formulations, and doses indicate daily doses

The study consisted of three phases: a prerandomisation phase; a maintenance phase in which double-blind treatment was carried out; and a follow-up phase (Figure 1). The study duration for each patient was up to 10 weeks and six visits (V1–6) were planned.

Following screening, patients entered either a titration or run-in period. Patients with inadequate pain control entered the titration period and were titrated and stabilised on a daily dose of oxycodone PR of 40, 60 or 80 mg. The starting oxycodone dose depended on previous pain medication. Patients already on stable oxycodone treatment with concomitant constipation, based on a clinical assessment referred to need for laxative intake to have three bowel movements per week, entered a 7-day run-in period and were eligible to enter the maintenance phase without prior titration. The oxycodone dose could be adjusted at any time during the titration or run-in period.

Patients who were receiving a stable oxycodone dose every 12 h at the end of the titration/run-in period, with no more than five rescue medication administrations per week, and who needed regular laxatives to have at least three bowel movements a week were randomised to three naloxone treatment groups or to placebo. Patients received their maintenance dose of oxycodone (given in an open-label fashion) plus a 10, 20 or 40 mg daily dose of naloxone PR or placebo (given in a double-blind manner) every 12 h for 4 weeks. Patients were advised to stop taking laxatives at the start of the maintenance phase, although they could be restarted if no bowel movements had occurred within 3 days. No dose adjustments were allowed during the maintenance phase. In the 2-week follow-up phase, patients received their maintenance dose of oxycodone PR without receiving naloxone PR.

The study was designed to evaluate the optimal dose ratio for oxycodone PR and naloxone PR. Within the three naloxone treatment groups, seven active oxycodone/naloxone dose ratios were evaluated (1 : 1, 1.5 : 1, 2 : 1, 3 : 1, 4 : 1, 6 : 1 and 8 : 1) (Table 1). Based on the results of the analgesic efficacy and bowel function presented elsewhere, the 2 : 1 dose ratio was deemed the optimal ratio for further development (15). For the global assessment, only data for the 2 : 1 dose ratio compared with the placebo group will be presented.

**Table 1:** Dose ratios

	Group 1	Group 2	Group 3	Group 4
Naloxone PR dailydose (mg)	Placebo	5 + 5	10 + 10	20 + 20
Oxycodone PRdaily dose (mg)	2 × 20, 2 × 30, 2 × 40	2 × 20, 2 × 30, 2 × 40	2 × 20, 2 × 30, 2 × 40	2 × 20, 2 × 30, 2 × 40
Oxycodone	20/placebo	20/10, 60/10, 80/10	40/20, 60/20, 80/20	40/40, 60/40
PR/naloxone	30/placebo			80/40
PR	40/placebo			
Dose ratio		2 : 1, 6 : 1, 8 : 1	2 : 1, 3 : 1, 4 : 1	1 : 1, 1.5 : 1, 2 : 1

In the placebo group, patients received oxycodone PR and a placebo. Whereas treatment groups received oxycodone PR plus naloxone PR 10, 20 or 40 mg/day. PR, prolonged-release.

The study outcome was global assessment of efficacy, tolerability and preference assessed at the end of the maintenance phase (V5). Safety assessments, including physical examination, standard laboratory tests and monitoring and recording of all adverse events were performed at each visit.

### Study assessments

Global assessment of efficacy and tolerability was completed at the end of the maintenance phase (V5) and rated independently by the investigators and the patients. The following rating scale was used: one = very good; two = good; three = fairly good; four = moderate; five = slightly poor; six = poor and seven = very poor.

Preference for the maintenance phase (oxycodone PR and naloxone PR) or the titration/run-in phase (oxycodone only) regarding tolerability and efficacy of study medication was also assessed at the end of the maintenance phase (V5). Preference was indicated using the following scale: one = titration/run-in; two = maintenance and three = no preference.

### Statistical analysis

For the global assessment of efficacy, tolerability and preference (assessed by the investigator and by the patient at the end of the maintenance phase), summary statistics for the 2 : 1 dose ratio of oxycodone and naloxone compared with placebo and absolute dose of naloxone were provided for the intention-to-treat (ITT) population with non-missing values. The percentages of patients rating the efficacy or tolerability of each treatment group as ‘good’ or ‘very good’ were combined to provide a composite positive score, while percentages for ‘moderate’, ‘slightly poor’, ‘poor’ and very poor’ were combined to give a negative score.

## Results

A total of 230 patients were screened; 202 were subsequently randomised and treated, and 166 completed the study. All randomised patients received study medication and were included in the safety population. The ITT population was defined as all randomised patients who received at least one dose of naloxone or corresponding placebo and who had at least one efficacy assessment, and consisted of 196 (97.0%) patients.

All treatment groups and dose ratio groups were well balanced in terms of demographics and baseline characteristics. No relevant differences were observed between treatment groups in terms of mean age, race, mean weight, mean height and mean body mass index. With regard to gender, 37.1% of patients in the study were male.

### Absolute naloxone PR dose

**Figure 2:**
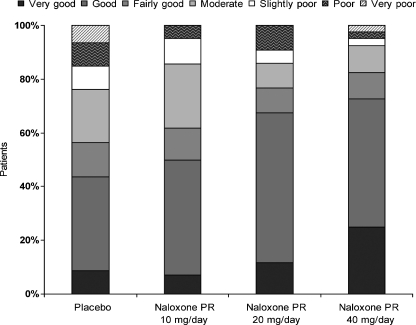
Patients’ global assessment of treatment efficacy at the end of the maintenance phase – relative frequencies by absolute naloxone prolonged-release (PR) dose (intention-to-treat population). In the placebo group, patients received oxycodone PR and a placebo. Whereas treatment groups received oxycodone PR plus naloxone PR 10, 20 or 40 mg/day

Patient global assessment of treatment efficacy improved with increasing naloxone PR dose. Efficacy was ranked as ‘good’ or ‘very good’ by 50.0%, 67.4% and 72.5% of patients in the 10, 20 and 40 mg/day naloxone PR dose groups respectively. In comparison, 43.5% of patients in the placebo group described efficacy as ‘good’ or ‘very good’ (Figure 2). The 40 mg naloxone PR dose was ranked as ‘moderate’ to ‘very poor’ by 17.5% of patients compared with 43.5% of patients who received placebo.

**Figure 3:**
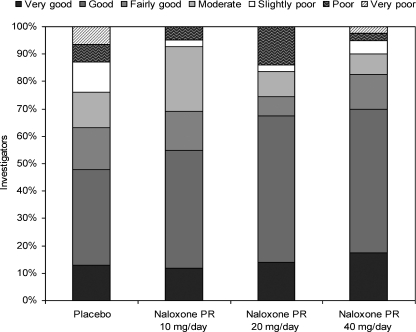
Investigators’ global assessment of treatment efficacy at the end of the maintenance phase – relative frequencies by absolute naloxone prolonged-release (PR) dose (intention-to-treat population). In the placebo group, patients received oxycodone PR and a placebo. Whereas treatment groups received oxycodone PR plus naloxone PR 10, 20 or 40 mg/day

This trend was mirrored by the investigators, with 54.8%, 67.4% and 70.0% ranking efficacy for the 10, 20 and 40 mg/day naloxone PR dose groups as ‘good’ or ‘very good’ respectively (Figure 3). Efficacy of the placebo group was ranked as being ‘good’ or ‘very good’ by 47.8% of investigators.

**Figure 4:**
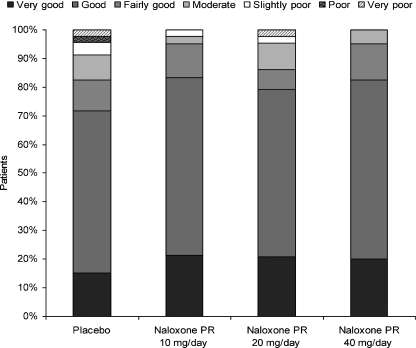
Patients’ global assessment of treatment tolerability at the end of the maintenance phase – relative frequencies by absolute naloxone prolonged-release (PR) dose (intention-to-treat population). In the placebo group, patients received oxycodone PR and a placebo. Whereas treatment groups received oxycodone PR plus naloxone PR 10, 20 or 40 mg/day

**Figure 5:**
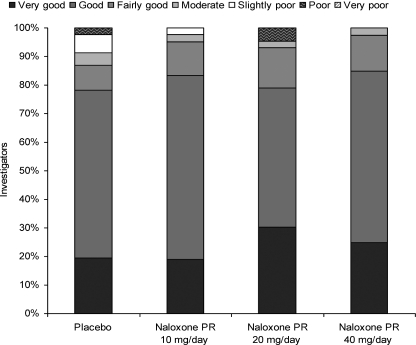
Investigators’ global assessment of treatment tolerability at the end of the maintenance phase – relative frequencies by absolute naloxone prolonged-release (PR) dose (intention-to-treat population). In the placebo group, patients received oxycodone PR and a placebo. Whereas treatment groups received oxycodone PR plus naloxone PR 10, 20 or 40 mg/day

Tolerability remained fairly stable with increasing naloxone dose. The tolerability of the 10 mg/day naloxone dose was ranked as ‘good’ or ‘very good’ by 83.3% of patients and investigators. The tolerability of the 20 mg/day naloxone dose was ranked as ‘good’ or ‘very good’ by 79.1% of patients and 79.1% of investigators. The 40 mg/day naloxone dose was ranked as ‘good’ or ‘very good’ by 82.5% of patients and 85.0% of investigators in terms of tolerability. This compared well with the tolerability of the placebo group, which was ranked as ‘good’ or ‘very good’ by 71.7% of patients and 78.3% of investigators (Figures 4 and 5).

**Figure 6:**
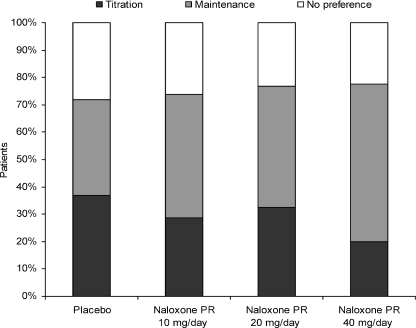
Patients’ preference of treatment efficacy according to study phase – relative frequencies by absolute naloxone prolonged-release (PR) dose (intention-to-treat population). In the placebo group, patients received oxycodone PR and a placebo. Whereas treatment groups received oxycodone PR plus naloxone PR 10, 20 or 40 mg/day

**Figure 7:**
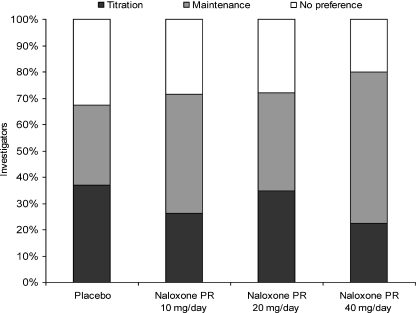
Investigators’ preference of treatment efficacy according to study phase – relative frequencies by absolute naloxone prolonged-release (PR) dose (intention-to-treat population). In the placebo group, patients received oxycodone PR and a placebo. Whereas treatment groups received oxycodone PR plus naloxone PR 10, 20 or 40 mg/day

The distribution of preference between ‘titration’, ‘maintenance’ and ‘no preference’ was generally even in terms of efficacy for patients in the placebo group – 37.0% of patients preferred the titration phase, 34.8% preferred the maintenance phase and 28.2% had no preference. Patient preference for the maintenance phase was 45.2%, 44.2% and 57.5% for the 10, 20 and 40 mg/day naloxone doses respectively (Figure 6). A similar trend towards preference for the maintenance phase was also observed with investigators (Figure 7).

With regard to the preference of treatment phase in terms of tolerability, a similar trend was observed. In the placebo group, 34.8% of patients preferred the maintenance phase compared with 54.8%, 60.5% and 57.5% of patients in the 10, 20 and 40 mg/day naloxone groups respectively. A similar trend was seen for investigators, with 34.8% preferring the maintenance phase in the placebo group compared with 52.4%, 55.8% and 60.0% for the 10, 20 and 40 mg naloxone groups respectively.

### Oxycodone PR/naloxone PR dose ratio

In terms of efficacy, the 2 : 1 dose ratio was ranked ‘good’ or ‘very good’ by 70.4% of patients and investigators. In comparison, placebo (40, 60 and 80 mg/placebo combined) was ranked as ‘good’ or ‘very good’ by 43.5% of patients and 47.8% of investigators. Only 18.5% of patients and 11.1% of investigators ranked the efficacy of the 2 : 1 dose ratio as ‘moderate’, ‘slightly poor’, ‘poor’ or ‘very poor’ compared with 37.0% and 43.5%, respectively, for placebo.

In terms of tolerability, the 2 : 1 dose ratio was ranked ‘good’ or ‘very good’ by 81.5% of patients and investigators. This compares favourably with placebo (40, 60 and 80 mg/placebo combined), which was ranked as ‘good’ or ‘very good’ by 78.3% of investigators and 71.7% of patients. None of the investigators and only 7.4% of patients ranked the tolerability of the 2 : 1 dose ratio as ‘moderate’ to ‘very poor’ compared with 13.0% and 17.4%, respectively, for placebo.

With regard to patient preference for treatment phase based on efficacy, the data for placebo were evenly distributed between the different study phases (titration phase 37.0%; maintenance phase 34.8%; no preference 28.3%). In contrast, the majority of patients in the treatment arm of the study preferred the maintenance phase in which they received oxycodone PR and naloxone PR. For the 2 : 1 dose ratio, 51.9% of patients preferred the maintenance phase, while 33.3% had no preference. A similar trend was seen for investigators, with 48.1% preferring the maintenance phase in the 2 : 1 dose ratio group compared with 30.4% who preferred the maintenance phase for the placebo group.

The distribution of patient preference between different treatment phases in the placebo group was also evenly distributed in terms of tolerability (titration phase 32.6%; maintenance phase 34.8%; no preference 32.6%). For the 2 : 1 dose ratio, 55.6% of patients preferred the maintenance phase, while 29.6% had no preference. A similar trend was seen for investigators, with 34.8% preferring the maintenance phase for the placebo group compared with 55.6% for the 2 : 1 dose ratio.

The safety analysis included a total of 202 patients. No trends or possible treatment-related pathological laboratory findings could be identified for any of the treatment groups. No deaths occurred during the study.

Adverse events during the maintenance phase were observed in all treatment groups (range: 62.7–70.0%), although the number of events increased with increasing naloxone PR dose, with 111, 119, 129 and 140 events in the placebo, 10, 20 and 40 mg/day naloxone PR dose groups respectively. Most adverse events were deemed to be mild or moderate in intensity based on investigator assessment. There was a slight trend for an increase in moderate and severe adverse events with increasing naloxone dose, but the incidence of serious adverse events was low and generally comparable across all active naloxone PR treatment groups. The most frequent adverse events were increased sweating, diarrhoea, nausea, abdominal pain, restlessness, muscle cramps, sedation, headache and vertigo.

## Discussion

The results from this clinical trial demonstrate that, in terms of patient assessment, the co-administration of oxycodone PR and naloxone PR is effective for the treatment of patients with severe chronic pain, whether cancer related or not. The study also indicates that increased naloxone PR dose (10, 20 or 40 mg/day) is associated with superior ratings of global assessment of efficacy and preference for treatment. Tolerability was similar for all doses of naloxone PR and placebo, indicating that the addition of naloxone does not cause further unwanted effects. While bowel function is classically seen as an issue related to opioid tolerability, in this study patients viewed the improvements in bowel function as part of the efficacy of the combination of oxycodone PR and naloxone PR.

The global assessment of preference shows that more patients preferred treatment during the maintenance phase, which consisted of the combination of naloxone PR and oxycodone PR, rather than the titration/run-in phase, which consisted of oxycodone PR only. This finding was mirrored by the investigators’ rating, which also showed a preference for maintenance phase of the study. In the oxycodone PR/placebo group, patient preference was relatively evenly distributed between the three response groups – maintenance phase, titration phase, no preference – and there was no overall preference for a specific response for either efficacy or tolerability. Again, this result was mirrored by the investigators’ assessment.

In conclusion, the addition of naloxone PR to oxycodone PR improves patient assessment of their analgesic therapy. This is especially significant for tolerability, where the stability of the patient preference across increasing naloxone dose indicates that the addition of naloxone to oxycodone does not result in any additional side effects. These results from the global assessment are confirmed by other results from this study presented elsewhere, which showed no impact of naloxone PR on the analgesic efficacy of oxycodone PR, with improvements in bowel function and reduced laxative intake (15).

Prevention of opioid-induced constipation is considered a more effective therapeutic strategy than treatment (11). The co-administration of oxycodone PR and naloxone PR has been shown to significantly reduce the impact of opioid-induced constipation, with dose-dependent increases in stool frequency and dose-dependent decreases in the use of laxatives (15).

Given the efficacy of oxycodone PR in a number of different pain syndromes, the addition of naloxone PR to prevent or reduce opioid-induced constipation can be of potential benefit to a significant number of patients suffering from chronic pain, allowing them to receive analgesia on a long-term basis and consequently to improve their quality of life. Indeed, the potential benefit of the oxycodone PR/naloxone PR combination is apparent by the patients’ preference for treatment during the maintenance phase during which they received both oxycodone PR and naloxone PR. Given the improvement in bowel function – and consequently quality of life – with the use of the oxycodone PR/naloxone PR combination, the management of severe, chronic pain will be facilitated for patients and physicians alike.
